# Rotational Gait Problems in the Presence of Femoral Deformity

**DOI:** 10.3390/bioengineering12111207

**Published:** 2025-11-05

**Authors:** Arik Rehani Musagara, Firooz Salami, Cornelia Putz, Nicholas A. Beckmann, Marco Götze, Sebastian I. Wolf

**Affiliations:** 1 Center for Orthopedics, Heidelberg University Hospital, 69118 Heidelberg, Germany; arik.rehanimusagara@gmail.com (A.R.M.); firooz.salami@med.uni-heidelberg.de (F.S.); cornelia.putz@med.uni-heidelberg.de (C.P.); nicholas.beckmann@med.uni-heidelberg.de (N.A.B.); marco.goetze@luks.ch (M.G.); 2 Children’s Hospital of Central Switzerland, Spitalstrasse, 6000 Lucerne, Switzerland

**Keywords:** femoral derotation osteotomy, trochanter prominence angle test, hip rotation, kinematics, functional calibration, knee joint axis

## Abstract

The relationship between femoral deformity and gait deviation is complex. Femoral anteversion can be assessed using the trochanter prominence angle test or by imaging techniques. Hip rotation during gait can be determined using conventional 3D gait analysis methods including palpation of femoral epicondyles or by using functional calibration. This study re-evaluates the indications for femoral osteotomies in this context. Hip rotation was analysed using predictive and functional methods in 80 patients who were referred for gait analysis due to rotational gait issues. Femoral anteversion was determined both manually and via MRI. In severe cases of femoral malalignment, the trochanter prominence angle test systematically underestimates the deformity by up to 15° compared to MRI results. Hip rotation, as measured by functional methods, also underestimates the outcome obtained by conventional methods, by up to 5°. Regardless of the method used, significant variability in hip rotation is observed during gait when the femoral deformation is moderate (anteversion between 0° and 30°). More severe deformities are not fully compensated for during gait. In cases of severe femoral malalignment, the functional change after osteotomy does not match the amount of derotation. Furthermore, both the trochanter prominence angle test and hip rotation during gait, as monitored via functional methods, underestimate the problem in the transverse plane.

## 1. Introduction

Rotational gait problems are a well-known symptom in orthopedics. They are typically treated with a femoral derotation osteotomy (FDO) especially when associated with increased femoral anteversion [1,2,3,4,5]. The aim is to reduce the risk of falling due to intoeing (i.e., feet crossing) and scissoring gait due to the internal orientation of the knee joints (i.e., knees crossing). In addition to structural findings (i.e., deformities) observed through manual testing [6] or imaging (CT or MRI) [7], as well as attempts to control the surgery via electromagnetic tracking methods [8], functional findings (i.e., the results of gait analysis) are also considered when planning an FDO and determining the optimal amount of derotation [5,9,10,11,12,13].

Several studies have shown that there is no straightforward correlation between structural and functional changes following femoral derotation osteotomy (FDO) [5,11,14,15]. Considering the limited precision of imaging and gait analysis data, as well as the limited precision with which the FDO can be performed, it appears that typically only around two-thirds of the amount of derotation of the femur is reflected in functional changes, i.e., hip rotation. With an FDO of 30°, approximately 10° of derotation appears to be ’lost’ when monitoring gait before and after surgery [15,16,17,18]. Therefore, we need to improve our understanding of how femoral deformities influence joint angles in the transverse plane during gait, and of how we quantify bony deformities and subsequent gait deviations.

In conventional gait analysis (CGA), hip rotation during gait is determined by monitoring the orientation of the knee joint axis (i.e., as palpated by the medial and lateral femoral epicondyles) in relation to the orientation of the pelvis in the transverse plane. A knee alignment device may be used to help place the respective markers onto the prominences of the knee [19]. We refer to this method as KAD. More recently, functional calibration methods have been developed to determine the orientation of the knee joint axis during movement [20]. This method is referred to as FUN. Subjects equipped with markers are asked to perform knee flexion/extension movements in either single-limb stance (unloaded) or bilateral squats (loaded). The average axis of flexion is then determined via optimization algorithms from the relative movement between the markers on the shank and thigh.

For typically developing (TD) subjects, it has been demonstrated that the KAD and FUN methods produce largely consistent results. The latter method is more precise and largely independent of the examiner, although it does depend somewhat on the choice and execution of the calibration movement [20,21]. However, in a group of 95 subjects presenting with varying hip rotation, we found that the results obtained via the KAD method for hip rotation do not agree with those obtained via the FUN method [22]. This is in contrast to TD subjects. Specifically, the larger the discrepancy from normal, the more the two methods disagree. For typical gait, the difference is negligible; however, for severe external or internal hip rotation, the deviation may be as large as 15°. Although less precise, the KAD method appeared to more accurately describe functional changes after FDO than the more recent FUN method, as evidenced by frontal plane video clips. In this study, knee rotation was considered to account for the systematic difference between the two methods. Therefore, it is unclear how to correctly determine rotational gait problems in the presence of femoral deformities, or how to measure femoral anteversion clinically [23].

This study aims to assess hip rotation during gait, as measured by the FUN and KAD methods, in a larger cohort of patients suspected of having a femoral deformity. Firstly, the study will seek to corroborate the evidence that these methods produce different results. Secondly, knee rotation will be determined by measuring the tibiofemoral angle in MRI scans in order to verify whether this parameter accounts for the difference in the respective functional gait measures. To quantify the underlying cause of gait deviation, femoral malalignment will be determined manually via the trochanteric prominence angle test (TPAT), and this measurement will be compared with MRI imaging for reference purposes, as it will relevantly influence surgical decision-making.

## 2. Materials and Methods

This study examined a total of 80 patients (mean age: 15.4 ± 5.1 years; mean BMI: 20.4 kg/m^2^; 50% male/50% female) retrospectively. Patients were included if they had been referred for gait analysis to evaluate potential rotational malalignment in gait. Each patient also had to provide an MRI scan (T1 sequence, axial slices taken by a 3-Tesla device, MAGNETOM Verio, Siemens, Forchheim, Germany) acquired within one month before or after the gait analysis in order to assess femoral anteversion. Patients who were unable to perform the required calibration trials, as described below, were excluded. Written informed consent was obtained from all participants according to the study protocol, which was approved by the local ethics committee (S-215/2019). Of the 80 patients, 22 were diagnosed with cerebral palsy (CP) (GMFCS I: 14, GMFCS II: 8), while the remaining 58 presented with idiopathic rotational malalignment.

According to the Plug-In Gait protocol [19] all participants were equipped with reflective skin-based markers, including additional thigh and shank markers for functional calibration assessment. See Musagara et al. [22] for more details. The optimal common shape technique [24] was applied to reduce the effect of soft-tissue artifacts (STAs). Three-dimensional (3D) motion data were captured using a 12-camera Vicon Nexus system (Vicon, Oxford Metrics, UK). An experienced examiner placed a knee alignment device (KAD) on each knee to determine the knee joint axis. For the functional approach, SARA [25] was implemented and is referred to here as ‘FUN’. Participants were asked to perform repetitive knee flexion and extension movements of the unloaded limb while standing on the other limb. They were instructed to perform the movement three times at a comfortable speed and range of motion. They were permitted to use assistive devices (e.g., crutches) or a helper’s hand to maintain balance during the calibration trials. Subsequently, the gait kinematics of at least seven strides at a self-selected speed were determined, and the outcome parameters mean hip and mean knee rotation in stance were calculated for both methods across all trials.

A physician with over five years’ experience analyzed the rotational MRI scans (T1 sequence axial slices taken by a 3-Tesla device, MAGNETOM Verio, Siemens, Forchheim, Germany) and determined the femoral anteversion in accordance with Koenig’s recommendations [26]. Further, the femorotibial angle was measured using the method described by Vassalou et al. [27]. Femoral anteversion was also measured clinically by applying the trochanter prominence angle test (TPAT) [6]. For this test, the patient lies prone with the hip in a neutral position and the knee flexed at 90°. The examiner palpates the greater trochanter on the side being tested and passively rotates the hip until the greater trochanter is in its most lateral and prominent position. The anteversion was then measured between the axis crossing the shaft of the tibia and a virtual line perpendicular to the table [28]. In our laboratory, all manual tests undergo quality assurance procedures. Patients are frequently tested independently by two examiners with more than five years’ professional experience. The repeatability is typically around 5–7°.

### Statistics

To avoid dependencies between legs, only the more severely affected leg of each patient was used for further analysis. Patients were divided into five groups according to anteversion as measured by MRI, with increments of 10° ranging from retroversion (<0°) to increased anteversion (>30°). Differences between groups were analyzed for all measured parameters using a one-way ANOVA and post hoc comparisons after Bonferroni correction. Additionally, hip and knee kinematics, as determined by conventional and functional methods were compared using Deming regression analysis [29]. Unlike a conventional linear method, this test allows for potential measurement errors within each method. Deming regression was also used to compare anteversion measured using transverse MR images and TPAT. The significance level was set at *p* < 0.05. All statistical analyses were performed using IBM SPSS 28 (IBM, Armonk, NY, USA) and XLSTAT (v. 2025.1, Addinsoft Inc., New York, NY, USA).

## 3. Results

**Figure 1:**
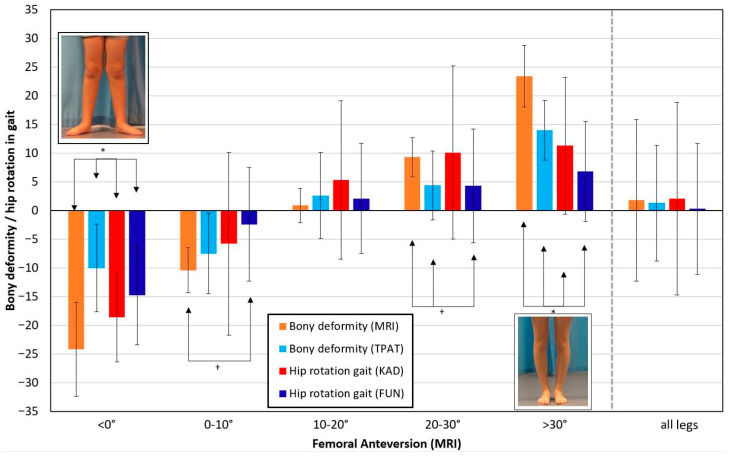
Legs are classified into subgroups according to femoral anteversion: <0°, 0–10°, 10–20°, 20–30°, and >30°, respectively. The bars represent the bony deformity as measured by MRI (orange) and by TPAT (light blue), as well as mean hip rotation according to KAD (red) and to FUN (dark blue). Standard deviations are shown for each group. Average data across all legs are shown to the right. The asterisk denotes statistically significant differences between all pairs within a group. The dagger denotes statistically significant differences between pairs as indicated by the arrows.

All 80 legs were classified into five subgroups according to MRI measurement, showing femoral anteversion of <0°, 0–10°, 10–20°, 20–30°, and >30°, respectively. The respective group sizes were 11, 16, 19, 17, and 17 legs. Figure 1 shows the deviation from the typical femoral anteversion angle of 15° [30] for each subgroup in. (Orange: bony deformity MRI; light blue: bony deformity TPAT). Furthermore, mean hip rotation in stance is shown (red: KAD; dark blue FUN). In the subgroup with a typical femoral anteversion angle of 10–20°, the measures largely match, with substantial variation in hip rotation (error bars). In cases of femoral retroversion (<0°) and in cases with severely increased femoral anteversion (>30°), the bony deformity according to TPAT is significantly underestimated compared to the MRI measures (*p* < 0.001 in both comparisons). Both functional measures (KAD and FUN) deviate similarly, albeit to a lesser extent (*p* = 0.045, and *p* = 0.046, respectively), with less variation in the FUN measures (error bars).

There was no significant variation in the femorotibial angle obtained by MRI or mean knee rotation during gait between subgroups. Knee rotation during gait was 3.8 ± 8.2° (KAD) and 5.3 ± 8.4° (FUN) across all groups, compared to 3.2 ± 5.6° for the femorotibial angle obtained by MRI.

**Figure 2:**
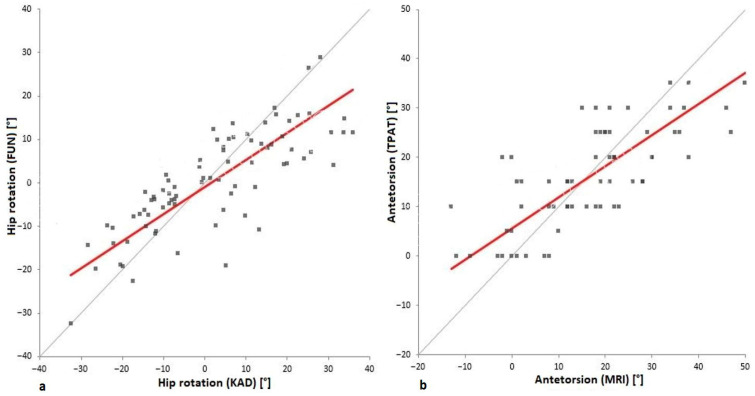
Linear regression (in red) according to Deming [29] was obtained for hip rotation in stance ((**a**); KAD versus FUN) and for femoral anteversion ((**b**); MRI versus TPAT). The additional thin grey line indicates unity and serves as a guide for the eye only.

Figure 2 shows the linear regressions obtained according to Deming between bony measures (MRI and TPAT) and between measures for mean hip rotation in stance (KAD and FUN) based on individual values. The functional measures (left diagram) coincide at 0° hip rotation (where the red regression line meets the grey identity line). Similarly, the bony measures coincide with typical femoral anteversion, i.e., at 15° (right diagram). The greater the deformity, the greater the discrepancy between TPAT and MRI, and the greater the deviation between the functional measures (KAD vs. FUN). The linear correlations in both comparisons are significant (*p* < 0.001) and show almost identical regression factors (0.63 for TPAT vs. MRI and 0.62 for FUN vs. KAD, respectively).

## 4. Discussion

Firstly, this study corroborates earlier findings [22] that the FUN and KAD measures of mean hip rotation during stance differ in the presence of a severe femoral deformity, with the FUN method largely underestimating the functional deviation (see Figure 1 for red and dark blue bars). Secondly, knee rotation, as measured by the tibiofemoral angle in MRI scans, does not explain the difference in the respective functional gait measures, as it remains rather constant across the groups sorted by femoral deformity. Thirdly, femoral malalignment, the underlying cause of gait deviation, is substantially underestimated in the manual TPAT, as can be seen by comparing it with MRI data (Figure 1, orange and light blue bars).

As shown in Figure 1, our results indicate that hip rotation measurements according to the FUN method lead to reduced external hip rotation when femoral anteversion is reduced (or when it is in retroversion), and vice versa. It also leads to less internal hip rotation when femoral anteversion is increased. However, the deviations of typically 3–6° are smaller than in our earlier work [22]. This may be partly due to the fact that there were fewer extreme cases in this cohort and that the underlying pathologies differed in number.

Another finding of this study is that although hip rotation (internal or external) during stance varies considerably within each subgroup, this variation is typically substantially smaller than the variation in bony deformation assessed via MRI. This variation may be due to variable compensation strategies or spasticity. This finding is consistent with an earlier result showing little correlation between bony deformation, as assessed by MRI, and hip rotation during walking [7]. However, Figure 1 shows, even when accounting for all variability, in cases of extreme bony deformity (i.e., femoral anteversion of less than 0° or more than 30°), the hip rotation angle during gait barely exceeds the amount of deformity (see error bars). It appears that there is a structural limit to the extent to which bony deformities can be compensated for during gait which may affect surgical decision-making in such extreme cases. Consequently, when the bony deformity is corrected surgically, the functional change in hip motion in the transverse plane during gait is usually less than the amount of femoral derotation. This explanation is consistent with the findings of the intervention studies [15,16,17,18].

In addition to the functional assessment of hip rotation during gait, the measurement of structural deformities is also subject to error. Although CT imaging is still considered the gold standard for measuring femoral anteversion, it has been demonstrated that the determination via MRI is only slightly inferior to it [31]. The interrater reliability (0.90) was almost identical to that determined using CT imaging (0.91), and the limits of agreement for repeated measurements (9.4°) were only slightly larger than those for CT imaging (8.2°), with a mean difference between the two methods (CT-MRI) of only 0.41°. Therefore, the choice of imaging technique (MRI) in this study does not account for differences to values of hip rotation during gait. However, substantial errors are present in the individual analysis (MRI and CT), and this must be considered when planning femoral derotation osteotomy.

Several previous studies have discussed the reliability of the TPAT, with results varying from poor to good outcomes [32,33]. There has also been critical discussion as to whether the method itself, which uses the tibia as a lever at a 90° knee flexion angle, might increase the knee joint space due to laxity of the knee joint [34]. Similarly, in our lab’s daily practice of performing the TPAT, we occasionally found that the knee anatomy was distorted, especially in cases with substantial femoral deformity. This meant that, when the patient was lying prone with the major trochanter positioned at its most lateral position, the axis given by the femoral epicondyles did not seem to be perpendicular to the long axis of the tibia. Therefore, the angle between the tibia and the bench may not be correct for femoral anteversion. Knee rotation, as measured via MRI with the knee extended, may be neglected in this test with the knee flexed at 90°. Considering the femoral epicondyle axis rather than the long axis of the tibia may provide a more accurate measurement of femoral anteversion. Yoon et al. [35] proposed this in 2014 under the name ‘transcondylar angle test’ (TCAT), using an electronic clinometer on a smartphone. Nevertheless, the TPAT is still frequently used in clinics and gait laboratories as it is a more time- and cost-efficient method of evaluating femoral anteversion than more complex imaging techniques. Consistent with studies that have questioned the validity of the TPAT [6,32,33,34,36], we found that its results were far more variable within subgroups than MRI measures (see error bars in Figure 1). Therefore, MRI measurements should clearly be given preference over manual testing, although an uncertainty of up to 10° should still be considered when planning femoral derotation.

Since both the TPAT and the determination of femoral anteversion via MRI are subject to significant error, we performed a Deming regression analysis [29] to check for systematic differences between the two methods. As Figure 2b shows, both measures produce similar results for typical anteversion (15°). However, for 40° anteversion, the systematic difference is approximately 15°, as reflected by the regression factor of 0.62. Somewhat surprisingly, Deming’s linear regression analysis of the two measures of hip rotation during gait (FUN vs. KAD) produced a very similar result: For typical gait, i.e., with neutral orientation of the hip, FUN and KAD produce the same result. However, for a 25° hip internal rotation according to KAD, the difference between the two measures is approximately 15°. The regression factor (0.63) is almost identical here.

Since we suspected that the knee had a systematic influence on both the measurement of femoral anteversion in the TPAT and the measurement of hip rotation during gait via FUN, we quantified additional knee parameters. However, neither the femorotibial angle, as measured by MRI, nor the knee rotation angle during gait (FUN and KAD) can account for such a systematic influence, since the respective angles remain relatively constant (within 2–8°) across subgroups and show significant individual variation (standard deviation: 8°).

In the inclusion criteria for this study, it was not considered whether patients had a neuromuscular disorder (i.e., CP) or not (i.e., idiopathic rotational malalignment). Amongst the 22 patients diagnosed with CP, only 5 patients showed mild hip external rotation during gait whereas 17 patients showed mild to severe hip internal rotation with typically increased femoral anteversion as the cause. In contrast, patients with idiopathic femoral malalignment more frequently showed external hip rotation during gait due to reduced femoral anteversion or retroversion. Hence, a detailed analysis of neuromuscular factors and compensation strategies is not possible. However, it should be noted, that the eleven patients with femoral retroversion (all idiopathic cases, left group in Figure 1) could largely compensate for the malalignment by hip rotation during gait (see in Figure 1 the difference of only 5° between orange and red bar). In the 17 patients with extreme anteversion (>30°) the compensation in gait is highly variable (see error bar for the red column on the right in Figure 1), for both idiopathic cases (10) and CP cases (7). Regardless of variability, the average difference between bony malalignment and hip rotation during gait is 12° in both subgroups. Therefore, this variable response to femoral malalignment in gait may not be due to neuromotor problems or spasticity.

## 5. Limitations

One limitation of this study is that the femoral anteversion angle was determined by a senior orthopedic surgeon who assessed the MR images only once. Therefore, the limits of agreement of around 10° for repeated measurements apply [31]. According to this study, CT imaging would have provided slightly greater precision. However, as this study was performed on juveniles, the standard procedure was to use MRI to ensure radiation safety.

Since the study is retrospective, potential selection bias in the patient group cannot be ruled out. Patients who were unable to perform repetitive knee flexion and extension movements for functional calibration had to be excluded, especially those with severe impairments due to spasticity.

## 6. Conclusions

The key finding of this study is that the hip joint does not usually compensate fully for the transverse plane deformity of the femur during gait in cases of femoral retroversion or anteversion of more than 30°. We consider this to be the dominant explanation for the finding in intervention studies that the functional change in gait is typically smaller than the derotation angle achieved through femoral osteotomy. MR imaging data (or CT data) should be used to assess femoral anteversion, rather than relying on the manual TPAT, since the latter method is highly variable and significantly underestimates severe femoral deformities. This should be considered when managing transverse plane deformities surgically. Similarly, the conventional method of assessing hip deviations in the transverse plane during gait (KAD) is preferable to the novel method (FUN), since the latter underestimates severe cases of internal rotation gait, despite showing better repeatability [22]. The study presented is unable to verify the hypothesis that the difference in outcomes between the two methods is caused by knee deformity.

## Data Availability

The original contributions presented in this study are included in the article. Further inquiries can be directed to the corresponding author.

## Abbreviations

The following abbreviations are used in this manuscript:

MRI	Magnetic Resonance Imaging
TPAT	Trochanter Prominence Angle Test
KAD	Knee Alignment Device
FUN	Functional Calibration
CGA	conventional gait analysis
TD	typical developing subjects
FDO	femoral derotation osteotomy
